# Supplementary material for: The causal role of circulating inflammatory markers in osteoporosis: a bidirectional Mendelian randomized study
Source: Front Immunol. 2024 Jul 18;15:1412298. doi: 10.3389/fimmu.2024.1412298 (PMC11291241; doi:10.3389/fimmu.2024.1412298)
Supplement: Supplementary file 1 [file DataSheet_1.zip › supplementary material/CIM-OSTEOPOROSIS /GCST90274836.csv_leave_one_out.pdf]

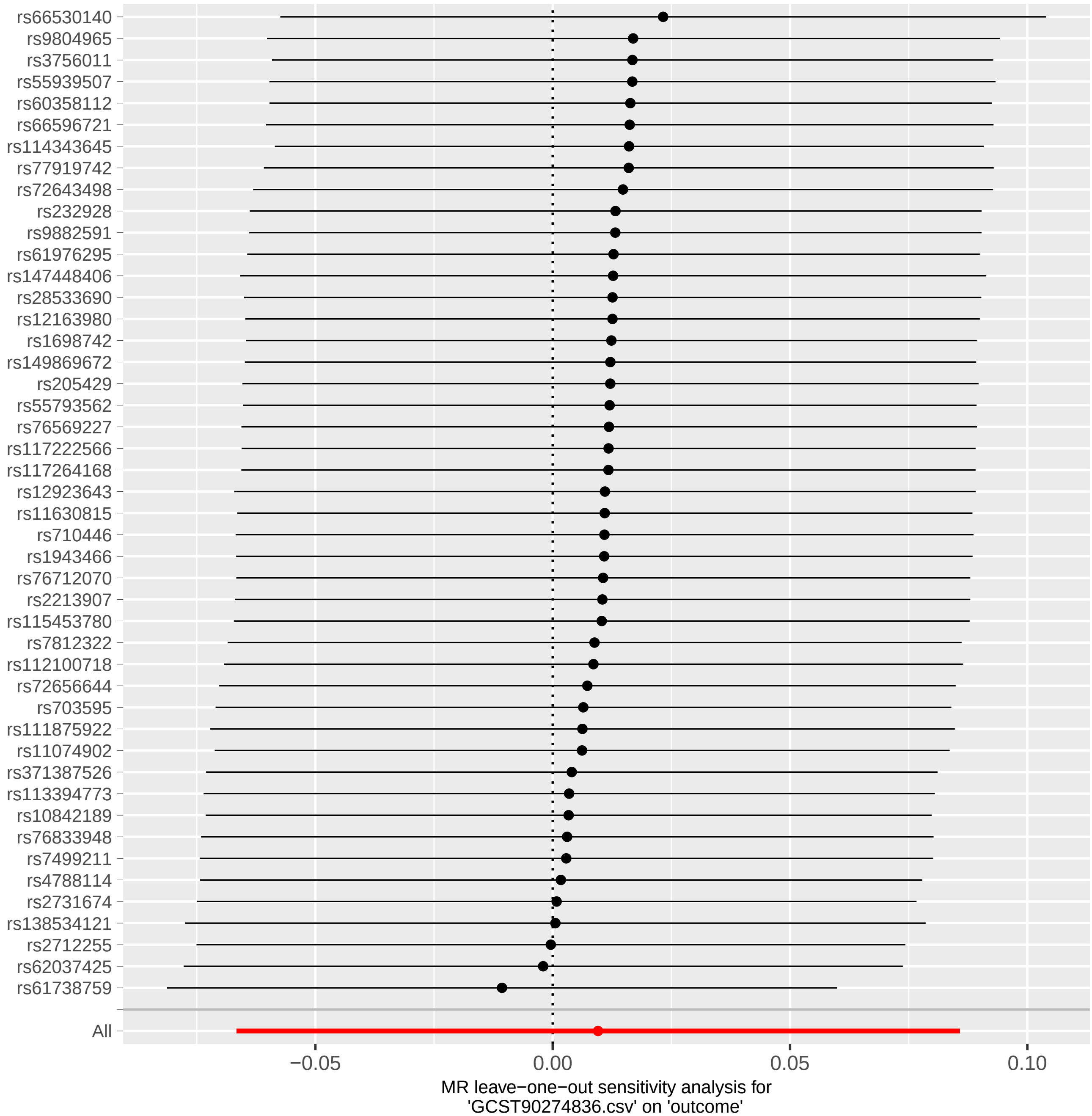

rs66530140
rs9804965
rs3756011
rs55939507
rs60358112
rs66596721
rs114343645
rs77919742
rs72643498
rs232928
rs9882591
rs61976295
rs147448406
rs28533690
rs12163980
rs1698742
rs149869672
rs205429
rs55793562
rs76569227
rs117222566
rs117264168
rs12923643
rs11630815
rs710446
rs1943466
rs76712070
rs2213907
rs115453780
rs7812322
rs112100718
rs72656644
rs703595
rs111875922
rs11074902
rs371387526
rs113394773
rs10842189
rs76833948
rs7499211
rs4788114
rs2731674
rs138534121
rs2712255
rs62037425
rs61738759
All
−0.05
0.00
0.05
0.10
MR leave−one−out sensitivity analysis for
'GCST90274836.csv' on 'outcome'
